# Polycomb protein SCML2 mediates paternal epigenetic inheritance through sperm chromatin

**DOI:** 10.1093/nar/gkad479

**Published:** 2023-06-07

**Authors:** Akihiko Sakashita, Masatoshi Ooga, Kai Otsuka, So Maezawa, Chikara Takeuchi, Sayaka Wakayama, Teruhiko Wakayama, Satoshi H Namekawa

**Affiliations:** Division of Reproductive Sciences, Division of Developmental Biology, Perinatal Institute, Cincinnati Children's Hospital Medical Center, Cincinnati, OH45229, USA; Department of Molecular Biology, Keio University School of Medicine, Tokyo160-8582, Japan; Faculty of Life and Environmental Science, University of Yamanashi, Kofu400-8510, Japan; Department of Microbiology and Molecular Genetics, University of California Davis, Davis, CA95616, USA; Department of Animal Science and Biotechnology, School of Veterinary Medicine, Azabu University, Sagamihara, Kanagawa252-5201, Japan; Department of Microbiology and Molecular Genetics, University of California Davis, Davis, CA95616, USA; Division of Reproductive Sciences, Division of Developmental Biology, Perinatal Institute, Cincinnati Children's Hospital Medical Center, Cincinnati, OH45229, USA; Department of Animal Science and Biotechnology, School of Veterinary Medicine, Azabu University, Sagamihara, Kanagawa252-5201, Japan; Faculty of Science and Technology, Department of Applied Biological Science, Tokyo University of Science, Chiba278-8510, Japan; Department of Molecular Biology, Keio University School of Medicine, Tokyo160-8582, Japan; Advanced Biotechnology Center, University of Yamanashi, Kofu400-8510, Japan; Faculty of Life and Environmental Science, University of Yamanashi, Kofu400-8510, Japan; Advanced Biotechnology Center, University of Yamanashi, Kofu400-8510, Japan; Division of Reproductive Sciences, Division of Developmental Biology, Perinatal Institute, Cincinnati Children's Hospital Medical Center, Cincinnati, OH45229, USA; Department of Microbiology and Molecular Genetics, University of California Davis, Davis, CA95616, USA

## Abstract

Sperm chromatin retains small amounts of histones, and chromatin states of sperm mirror gene expression programs of the next generation. However, it remains largely unknown how paternal epigenetic information is transmitted through sperm chromatin. Here, we present a novel mouse model of paternal epigenetic inheritance, in which deposition of Polycomb repressive complex 2 (PRC2) mediated-repressive H3K27me3 is attenuated in the paternal germline. By applying modified methods of assisted reproductive technology using testicular sperm, we rescued infertility of mice missing Polycomb protein SCML2, which regulates germline gene expression by establishing H3K27me3 on bivalent promoters with other active marks H3K4me2/3. We profiled epigenomic states (H3K27me3 and H3K4me3) of testicular sperm and epididymal sperm, demonstrating that the epididymal pattern of the sperm epigenome is already established in testicular sperm and that SCML2 is required for this process. In F1 males of X-linked *Scml2*-knockout mice, which have a wild-type genotype, gene expression is dysregulated in the male germline during spermiogenesis. These dysregulated genes are targets of SCML2-mediated H3K27me3 in F0 sperm. Further, dysregulation of gene expression was observed in the mutant-derived wild-type F1 preimplantation embryos. Together, we present functional evidence that the classic epigenetic regulator Polycomb mediates paternal epigenetic inheritance through sperm chromatin.

## Graphical Abstract

**Graphical Abstract:**
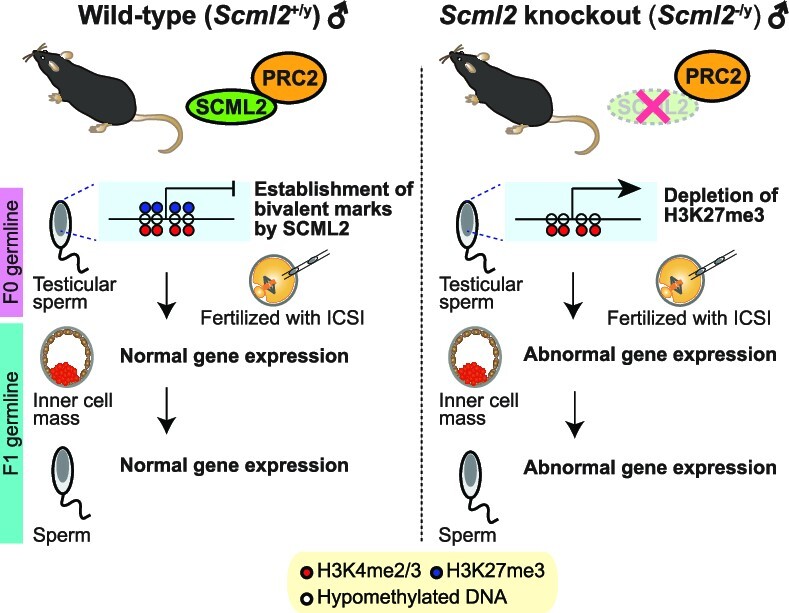

## INTRODUCTION

Emerging studies have established that the germline transmits epigenetic information to the next generation. In the male germline, paternal epigenetic states are subject to environmental and metabolic perturbation, carried through compacted sperm and impacting gene expression and disease risks in the next generation (1–5). In many cases, paternal epigenetic states have an intergenerational impact and are largely reprogrammed in the germline, though the extent of transgenerational inheritance across multiple generations remains debated (6,7). Several mechanisms are known to be responsible for intergenerational transmission of epigenetic states, including histone modifications on retained histones, DNA methylation, and small non-coding RNA (8–11). In particular, while the vast majority of histones are replaced with protamine in spermiogenesis, a small portion of histones are retained on gene regulatory elements in sperm; the chromatin states of sperm mirror the gene expression patterns of the next generation (12–19). However, the functions of sperm chromatin remain largely undetermined.

A prominent epigenetic signature of the mammalian germline is bivalent genomic domains, characterized by concomitant enrichment in repressive Polycomb-mediated trimethylation of histone H3 at lysine 27 (H3K27me3) and active di/trimethylation of histone H3 at lysine 4 (H3K4me2/3) marks on gene promoters, which persists in mature sperm (12,13,15,20–22). Bivalent domains represent molecular hallmarks of developmental potential in embryonic stem cells (ESC) (23–25). Thus, in the germline, bivalent domains are considered to be mediators of epigenetic inheritance to the next generation (26–28). In late spermatogenesis, bivalent domains became prevalent, covering thousands of genes that are activated later in the next generation (27–29). Of note, H3K4me3 is transmitted from sperm to embryos and is associated with embryonic gene expression (30). Overexpression of H3K4me2 demethylase KDM1A in the germline perturbs inter-and transgenerational inheritance of paternal epigenetic states and offspring health (30,31), but its effect is independent of bivalent domains (32). On the other hand, paternal deletion of H3K27me3 demethylase KDM6A leads to epigenetic inheritance and increases cancer susceptibility of the next generation (33). Despite this progress, the function of H3K27me3 remains to be tested in paternal epigenetic inheritance due to the lack of mouse models, in which H3K27me3 is attenuated in the male germline.

Polycomb group proteins suppress non-lineage specific genes and define cellular identity during development (34,35). Mammalian Polycomb proteins comprise two functionally related major complexes—Polycomb repressive complex 1 (PRC1) and PRC2—that catalyze monoubiquitination of histone H2A at lysine 119 (H2AK119ub) and H3K27me3, respectively (36). We identified a germline-specific Polycomb protein, SCML2, as a critical regulator of germline transcriptomes in the later stages of spermatogenesis (37–40). SCML2 interacts with both PRC1 and PRC2 and coordinates their activities to establish PRC2-mediated H3K27me3 and the bivalent domains in late spermatogenesis (29,37). SCML2 binding sites in spermatogonia predict the sites of H3K27me3 deposition in late spermatogenesis; loss of SCML2 resulted in H3K27me3 depletion and defective bivalent domains (29). Furthermore, *Scml2*-knock out (*Scml2*-KO) mice were infertile, and *Scml2*-KO epididymal sperm have abnormal shape (37), with midpieces tightly associated with sperm nuclei (29). The function of H3K27me3 in paternal epigenetic inheritance could not be determined in our previous studies (29,37). In this study, by applying modified methods of assisted reproductive technology (ART) using testicular sperm, we were able to rescue the male infertility of *Scml2*-KO mice. We performed native ChIP-seq of H3K27me3 and H3K4me3 in testicular sperm and epididymal sperm, demonstrating that the epididymal pattern of sperm epigenome is already established in testicular sperm and that SCML2 is required for this process. Here, we determine the paternal epigenetic inheritance mediated by SCML2 through sperm chromatin.

## MATERIALS AND METHODS

### Animals

Mice were maintained and used according to the guidelines of the Institutional Animal Care and Use Committee (protocol no. IACUC2018–0040) at Cincinnati Children's Hospital Medical Center and (reference number: A29-24) Yamanashi University. The founder *Scml2*^+/–^ female was generated in the C57BL/6 background using zinc finger nuclease technology (37). *Scml2*^+/–^ females were maintained by mating with C57BL/6 male mice. Therefore, *Scml2*-KO males (*Scml2*^-/y^) have a pure C57BL/6 background. Through mating between wild-type C57BL/6 males and *Scml2*^+/−^ females, *Scml2*-KO^(-/y)^ male mice were born at expected ratios according to Mendel's Law. For the genotyping of *Scml2*-KO mice, PCR was carried out using specific primer sets (37). Since *Scml2*-KO male mice showed infertility with abnormal sperm morphology, F1 generations from littermate wild-type and *Scml2*-KO males were generated using ART (intracytoplasmic sperm injection (ICSI) and round spermatids injection (ROSI)). To generate F2 mice, single F1 male progenies were continuously housed with 1-to-3 C57BL/6 females, and litters were weaned at 3 weeks of age.

### Collection of testicular spermatozoa and round spermatids for ICSI and ROSI

To obtain testicular spermatozoa and round spermatids, a single cell suspension from whole adult testis (wild-type and *Scml2*-KO) was prepared as described (29). Briefly, individual testes from wild-type and *Scml2*-KO mice at 8–12 weeks of age were dissected with fine forceps using sterile procedures in a 35-mm Petri dish containing 1× Enriched Krebs–Ringer bicarbonate (EKRB, containing 2.12 g/l sodium bicarbonate, Sigma) buffer, supplemented with 1× GlutaMax (Thermo Fisher Scientific), 1× non-essential amino acids (Thermo Fisher Scientific), 1× essential amino acid (Thermo Fisher Scientific), 1.3 mM calcium chloride, 1.2 mM magnesium sulfate and 1× penicillin–streptomycin (Thermo Fisher Scientific). After removing tunica albuginea membrane and unraveling seminiferous tubules, tubules were digested with collagenase (5 mg/ml, Sigma) at 35°C for 20 min with gentle pipetting every 5 min and then centrifuged at 188 xg for 5 min. The pellets were washed with the 1× EKRB buffer and then digested with trypsin (2.5 mg/ml, Sigma) at 35°C for 15 min. After incubation, Soybean trypsin inhibitor (50 μg/ml, Sigma) was added to neutralize the trypsin. Cells were filtered with a 40-μm strainer and resuspended with 1 ml of CELLBANKER 1 (Zenogen Pharma). The Cryopreserved testicular cells were stored in liquid nitrogen until use. For ICSI and ROSI, testicular spermatozoa and round spermatids were selected under the microscope based on their morphological features. These sperm nuclei were detached from midpieces after cryopreservation, prior to ICSI. This was not possible in our previous ICSI experiments using *Scml2*-KO fresh epididymal sperm, since epididymal sperm were surrounded by their midpieces that were tightly associated with nuclei (29). We were not able to dissociate midpieces from sperm nuclei, which might have prevented ICSI from working in our previous study (29).

### ICSI and ROSI

**Figure 1.**
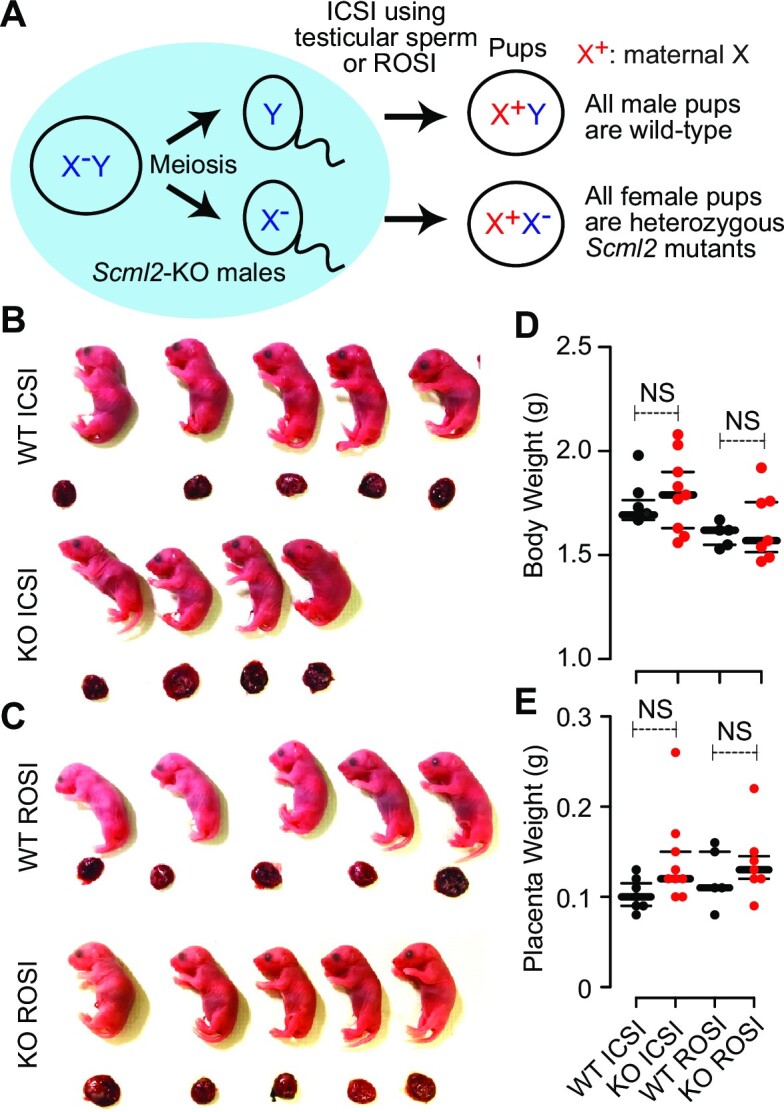
ICSI using testicular sperm or ROSI rescued infertility of *Scml2*-KO males. (**A**) Schematic of ICSI and ROSI using *Scml2*-KO males. (**B**, **C**) Representative images of full-term live offsprings and placentas produced from *Scml2*-KO (KO) and wild-type (WT) control testicular spermatogenic cells (ICSI using testicular elongated sperm or ROSI using round spermatids). (**D**, **E**) Body and placental weights of the live offsprings from each genotype at natural parturition or caesarean section. NS = not significant. Central bars represent medians; the small bars encompass 50% of the data points.

Oocytes and cumulus cells complexes were obtained from BDF1 female mice (SLC, Shizuoka, Japan) for Figure 1 and Supplementary Table S1 and from C57BL/6 female (SLC, Shizuoka, Japan) for Figure 7 and Supplementary Table S2, which had been injected with 7.5 IU of equine chorionic gonadotropin (ASKA Pharmaceutical Co., Ltd, Tokyo, Japan) and human chorionic gonadotropin (ASKA Pharmaceutical). Obtained oocytes and cumulus cells complexes were treated with hyaluronidase for 10 min; then the oocytes were retrieved. The cryopreserved testicular pellet was washed two times with D-MEM supplemented with 10% fetal bovine serum. After washing, the pellet was transferred to 10% PVP containing HEPES-buffered CZB-medium (CZB-HEPES) (41). Prior to cytosolic injection, zona pellucida and cytosolic membrane were disrupted with a piezo drive micromanipulator (Prime Tech Ltd, Ibaraki, Japan). Round spermatids or testicular spermatozoa were injected into the oocytes in CZB-HEPES. Ten min after injection, oocytes were washed and cultured in CZB-HEPES for 10 min. To obtain higher activation rate, 10 min after culturing in CZB-HEPES, round spermatid-injected oocytes as well as spermatozoa-injected oocytes were subjected to activation by culturing in Ca^2+^-free CZB-HEPES containing 5 mM SrCl_2_ for 1–2 h.

### Embryo culture and transfer

ICSI and ROSI-derived embryos were cultured in droplets of CZB-HEPES under paraffin oil in a 5% CO_2_ atmosphere at 37.5°C, until the desired developmental stage. To obtain full-term live offsprings, on the following day of ICSI and ROSI, the embryos that reached the two-cell stage were transferred into the oviducts of pseudopregnant female ICR mice at 0.5 days post-coitum (dpc), which had mated with a vasectomized ICR male overnight on the day before embryo transfer. At 18.5 dpc, the pups were obtained by the caesarean section (though some ones were delivered by natural parturition) and nursed by foster mothers. For the RNA-seq experiments in Figure 7, ICSI-derived embryos were kept in culturing for 4 days until they became blastocysts. 

### RNA-seq

Total RNA from the liver tissue was extracted with TRIzol reagent (Thermo Fisher Scientific) and purified using an RNeasy Plus Mini Kit (QIAGEN) with genomic DNA elimination according to the manufacturer's instructions. RNA-seq library preparation was carried out using a TruSeq Stranded mRNA Library Prep Kit (Illumina), following the manufacturer's instructions. For the preparation of the sperm RNA-seq library, we extracted total RNA from the swim-up separated motile sperm from the cauda epididymis using an RNeasy Plus Mini Kit (QIAGEN) with genomic DNA elimination according to the manufacturer's instructions. cDNA synthesis and preamplification were performed with total RNA using a SMART-Seq v4 Ultra Low Input RNA Kit and an Advantage 2 PCR Kit (Clontech), respectively. Preamplified cDNAs were supplied for RNA-seq library preparation with Nextera XT Library Prep Kit (Illumina). Indexed libraries were pooled and sequenced using an Illumina HiSeq-4000 or NextSeq-500 sequencer (single-end, 75 bp). Two independent biological replicates were generated for each sample.

The protocol for the construction of the embryo total RNA-seq libraries was adapted from a previous report with minor modifications (42). Briefly, the ICSI-derived two-cell and blastocyst embryos sired by wild-type control (Ctrl) and *Scml2*-KO sperm were treated with Acidic Tyrode's solution (Sigma) containing 0.5% polyvinylpyrrolidone and 50 mM NaCl to remove the zona pellucida (ZP) from embryos before sample collection, and then washed with PBS containing 0.2% polyvinyl alcohol. Pooled 30 each of ZP-free two-cells per replicate, and every single blastocyst in Ctrl-F1 and *Scml2*-KO-F1 embryos were lysed in 1× Lysis Buffer containing RNase inhibitor (0.2 IU/μl, from SMART-Seq Stranded Kit, Clontech), directly and stored at -80°C until subsequent library preparation. For PCR-based sexing, the lysates of every single blastocyst were boiled at 95°C for 10 min and subjected to PCR with KOD One polymerase (TOYOBO) and a specific primer set: *Zfy1*_Fw (5′-GACTAGACATGTCTTAACATCTGTCC-3′) and *Zfy1*_Rv (5′-CCTATTGCATGGACAGCAGCTTATG-3′). PCR was carried out using the following conditions: 95°C for 2 min, followed by 40 cycles each of 98°C for 10 s and 68°C for 30 s, on a ProFlex PCR System (Thermo Fisher Scientific). PCR products were visualized with electrophoresis on a 2% TAE agarose gel, and the genetic sex of each blastocyst was determined by the presence of PCR products. According to the manufacturer's instructions, the total RNA-seq libraries of pooled two-cell and single XY blastocyst embryos were prepared using SMART-Seq Stranded Kit (Clontech). RNAs were randomly sheared by heating at 85°C for 6 min and subjected to reverse transcription with random hexamers and PCR amplification. Ribosomal fragments were depleted from each cDNA sample with scZapR and scR-Probes. Indexed total RNA-seq libraries were enriched through a second PCR amplification and sequenced using an Illumina HiSeqX sequencer (paired-end, 150 bp).

### RNA-seq analysis

RNA-seq analysis was performed as described previously (43). Raw single-end RNA-seq reads from F1-liver, F1-sperm and F2-sperm samples were aligned to the mouse genome (GRCm38/mm10) using Hisat2 version 2.1.0 (44) with default settings. Alternatively, raw paired-end RNA-seq reads from two-cell and blastocyst embryos sired by Ctrl and *Scml2*-KO sperm, were aligned to mouse genome (GRCm38/mm10) using STAR aligner version 2.5.3a (45) with following options, - -twopassMode Basic; - -outSAMtype BAM SortedByCoordinate; - -outFilterType BySJout; - -outFilterMultimapNmax 1; - -winAnchorMultimapNmax 1; - -alignSJoverhangMin 8; - -alignSJDBoverhangMin 1; - -outFilterMismatchNmax 1; - -outFilterMismatchNoverReadLmax 0.04; - -alignIntronMin 20; - -alignIntronMax 1000000; - -alignMatesGapMax 1000000 for unique alignments. To visualize read enrichments over representative genomic loci, coverage files in TDF and bigWig formats were created from sorted BAM files using the IGVTools count function (Broad Institute) and bamCoverage program as implemented in deepTools (version 3.1.3) (46). Figures of continuous tag counts over selected genomic intervals were created in the IGV browser (Broad Institute). For in silico genotyping of each single blastocyst, we manually checked the coverage tracks of RNA-seq reads on Y chromosome, and then excluded dataset (XX blastocysts) in which we observed almost no aligned reads to Y chromosome. To quantify uniquely aligned reads on respective annotated gene transcript and repetitive loci, the featureCounts function, part of the Subread package (33), was used. The RPKM expression levels for each transcript were calculated using StringTie version 1.3.4 (47). Pearson correlation coefficients between biological replicates of RNA-seq profiles were calculated using SeqMonk (Babraham Bioinformatics). The gene expression matrix of RNA-seq datasets was applied to bidimensional PCA with R programming. Each sample score from the covariance matrix was plotted in the two eigenvectors PC1 and PC2 with ggplot2 (https://github.com/tidyverse/ggplot2). To detect differentially expressed genes between two biological samples, a read count output file was input to the DESeq2 package version 1.16.1 (48); program functions DESeqDataSetFromMatrix and DESeq were then used to compare each gene's expression level between two biological samples. Differentially expressed genes were identified through two criteria: (i) ≥2-fold change and (ii) binominal tests (*P*_adj_ < 0.01; *P* values were adjusted for multiple testing using the Benjamini-Hochberg method, or *P* < 0.01). To perform gene ontology analysis of gene sets, differentially expressed between two biological samples, the functional annotation clustering tool in DAVID version 6.8 (49) and Enrichr (50) were used, and a background of all mouse genes was applied. Biological process term groups with a *P* < 0.05 (modified Fisher's exact test) were considered significant. Further analysis was performed with R (version 3.4.0) and visualized as heat maps using Morpheus (https://software.broadinstitute.org/morpheus, Broad Institute).

### Collection of sperm from the testis and cauda epididymis for native ChIP-seq

For testicular sperm collection, tunica albuginea were removed, and seminiferous tubules were physically dissociated with tweezers. Tubules were further dissociated by 10 ml of EKRB Buffer, supplemented with 400 μg/ml Collagenase (Worthington Biochemical Corp., NJ), 80 μg/ml Hyaluronidase (Sigma), and 1.6 μg/ml DNase (Sigma), at 35°C for 10 min. Tubules were then centrifuged at 100 g at room temperature for 5 min. Then, the supernatant fraction was further centrifuged at 200 g at room temperature for 5 min. This supernatant fraction was filtered with a 25-μm mesh and centrifuged at 2500 rpm at room temperature for 5min. The pellet was used as testicular sperm for native ChIP-seq.

For epididymal sperm collection, cauda epididymides were transferred in a 5% CO_2_-equillibrated M2 medium (Sigma). Each epididymis was thoroughly pierced with a 27G needle and incubated at 37°C in a 5% CO_2_ condition for 30 min. M2 medium containing the swim-up sperm was collected carefully and filtered with a 25-μm mesh. Filtered sperms were centrifuged at 200 g at room temperature for 5 min, and the supernatant was further centrifuged at 2500 rpm at room temperature for 5min. The pellet was used as epididymal sperm for native ChIP-seq.

### Sperm native ChIP-seq

Sperm native ChIP-seq was performed by following a previously published protocol (51) with minor modifications. Sperm (2.6–4.9 × 10^5^ cells per sample) were suspended in PBS and treated with 50 mM DTT at room temperature for 2 h. DTT was quenched by 100 mM *N*-ethylmaleimide. After washing with PBS, sperm were treated with a buffer, containing 15 mM Tris–HCl (pH 7.5), 60 mM KCl, 5 mM MgCl_2_, 0.1 mM EGTA, 1% NP-40, and 1% sodium deoxycholate, on ice for 10 min. MNase treatment was performed using detergent-treated sperms with 6000 gel units of MNase (New England Biolabs, MA) per sample at 37°C for 5 min and was immediately inhibited by 5 mM EDTA. After MNase treatment, 10% of total chromatin was separated and used as an input sample. Sperm chromatin was first pre-cleared with protein A/G (1:1) Dynabeads (Life Technologies, CA), then reacted with 1 μl of anti-H3K27me3 antibody (C15410195, Diagenode, Belgium) or anti-H3K4me3 antibody (39155, Active Motif, CA) at 4°C overnight with gentle rotation. For antibody-beads conjugation, protein A/G Dynabeads were added, and samples were incubated at 4°C for 4 h with gentle rotation. After incubation, to remove non-specific bound chromatin, samples were washed once with a low-salt washing buffer, containing 50 mM Tris–HCl (pH 7.5), 10 mM EDTA and 75 mM NaCl, and twice with a high-salt washing buffer, containing 50 mM Tris–HCl (pH 7.5), 10 mM EDTA and 125 mM NaCl. ChIP sample was then eluted with 1% SDS/PBS, treated with 200μg/ml of RNase A (Thermo Fisher Scientific) at 37°C for 30 min and further treated with 200 μg/ml of Proteinase K (Invitrogen, CA) at 55°C overnight. Finally, DNA was purified with phenol/chloroform/isoamyl alcohol and precipitated with ethanol.

**Figure 2.**
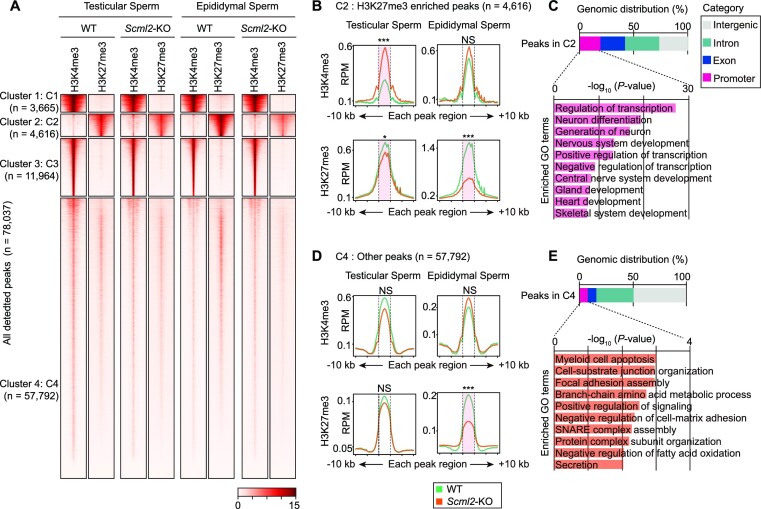
H3K27me3 and H3K4me3 distributions in testicular sperm and epididymal sperm. (**A**) k-means clustered heatmap of all detected peaks (detected by MACS2) of H3K4me3 and H3K27me3 in wild-type (WT) and *Scml2*-KO testicular sperm and epididymal sperm. (B, D) Average tag density plots around each peak region ±10 kb for Cluster 2: C2 (**B**) and for Cluster 4: C4 (**D**). *P < 0.05; ***P < 0.001; NS, not significant; Wilcoxon rank-sum test with Bonferroni correction was performed on each peak region (shown with dotted lines). (C, E) Genomic distribution of peaks in Cluster 2: C2 (**C**) and in Cluster 4: C4 (**E**) and bar charts showing enriched GO terms for genes that have promoter peaks.

ChIP-seq library preparation was carried out using a NEBNext Ultra II DNA Library Prep Kit (New England Biolabs) according to the manufacturers’ instructions. Indexed libraries were pooled and sequenced using an Illumina Hiseq X sequencer (paired-end, 150 bp). Two independent biological replicates were generated for each sample. For the analysis of Figures 2, 4, 5 and Supplementary Figure S3, we analyzed representative data sets (all data for rep1 except for wild-type epididymal sperm H3K4me3 and H3K27me3: rep2).

### ChIP-seq analyses

Raw paired-end ChIP-seq reads were aligned to the mouse genome (GRCm38/mm10) using bowtie2 version 2.3.3.1 with default settings (52); the reads were filtered to remove alignments mapped to multiple locations by calling grep with the -v option. Peak calling for ChIP-seq data was performed by MACS2 version 2.1.4 with default arguments (53); a cut-off *P*-value of 10^−2^ was used. Relative ChIP-seq enrichments in respective loci were calculated by dividing input controls. The program ngs.plot (54) was used to draw tag density plots and heatmaps for H3K4me3, H3K27me3, H3.3, and H3.1/H3.2 ChIP-seq read enrichment within ±10 kb of TSS. For k-means clustering of all H3K4me3 and H3K27me3 peaks in testicular and epididymal sperm from wild-type and *Scml2*-KO males, we firstly generated a set of regions that were called peaks with MACS2 in at least one of the datasets, by merging peak regions of all biological replicates using the mergedBed function from BEDTools (version 2.30.0) (55). Using respective merged peak files from H3K4me3 and H3K27me3 ChIP-seq data, we ran k-means clustering analysis using computeMatrix and plotHeatmap program as implemented in deepTools (version 3.1.3) (46) and determined that *k* = 4 was suitable for our data. To evaluate the functional annotation of each cluster, we utilized Genomic Region Enrichment of Annotation Tool (GREAT, version 4.0.4) (56) and HOMER (version 4.9) (57), both of which associates ChIP-seq peak regions in each cluster with their genomic feature and ontology of their putative target genes adjacent to peaks. To visualize read enrichment over representative genomic loci, coverage files in TDF and bigWig formats were created from sorted BAM files using the IGVTools count function (Broad Institute) and bamCoverage program as implemented in deepTools (version 3.1.3) (46). Figures for continuous tag counts over selected genomic intervals were created in the IGV browser (Broad Institute). Pearson correlation coefficients between biological replicates of ChIP-seq profiles (bin = 10 kb) were calculated using SeqMonk (Babraham Bioinformatics).

## RESULTS

### ICSI or ROSI rescued infertility of *Scml2*-KO mice

Because *Scml2* is an X-linked gene, all-male pups sired from *Scml2*-KO males are wild-type, while all-female pups are heterozygous *Scml2* mutants (Figure 1A). Therefore, *Scml2*-KO mice are an ideal model to study epigenetic inheritance from the paternal germline to the descendant male germline without transmission of the mutant allele. To test the functions of SCML2 in paternal epigenetic inheritance, we sought to recover the infertility of *Scml2*-KO mice by ART. Because our previous attempts of *in vitro* fertilization (IVF) or ICSI using *Scml2*-KO epididymal sperm with wild-type oocytes were not successful (29), we tried a modified method of ICSI using testicular sperm that had not yet undergone later maturation processes. Notably, ICSI using testicular sperm rescued infertility of *Scml2*-KO males, and these pups underwent full-term development with apparently normal placentas at a comparable frequency with that of controls (Figure 1B, Supplementary Table S1). As an alternative approach, we further performed ROSI using *Scml2*-KO round spermatids and recovered pups with full-term development at the comparable frequency with that of controls (Figure 1C, Supplementary Table S1). Body weight and placental weight were similar between wild-type- and *Scml2*-KO-derived pups from ICSI or ROSI (Figure 1D and E). Consistently, *Scml2*-KO-derived embryos normally reached the two-cell stage prior to the transfer to pseudopregnant surrogate females (Supplementary Table S1). In this study, we focused on *Scml2*-KO-derived males sired by ICSI as a model of epigenetic inheritance.

### H3K27me3 and H3K4me3 distribution in testicular and epididymal sperm

Since we were able to rescue the infertility of *Scml2*-KO male with ICSI using testicular sperm, we sought to determine how the sperm epigenome is regulated in testicular sperm prior to its maturation to epididymal sperm. Although many previous studies have reported the epigenomic status of epididymal sperm in mice (12–19), the epigenomic status of testicular sperm has never been reported. To test whether epigenomic states alter from testicular sperm to epididymal sperm and whether SCML2 is required for this process, we examined distributions of H3K4me3 and H3K27me3 in testicular sperm and cauda epididymal sperm isolated from wild-type and *Scml2*-KO males by native ChIP-seq experiments. We confirmed nearly 100% purity of our isolated sperm fractions (Supplementary Figure S1) and reproducibility of our native ChIP-seq experiments of H3K4me3 and H3K27me3 in testicular sperm and cauda epididymal sperm between biological replicates (Supplementary Figure S2). We detected 78037 peaks among all samples, categorized into 4 clusters through k-means clustering (Figure 2A). Cluster 1 and 3 peaks show enrichment of H3K4me3, while Cluster 2 peaks are enriched with H3K27me3 but modestly marked with H3K4me3. Cluster 4 peaks are marked with both H3K4me3 and H3K27me3. Overall, the distribution patterns of H3K4me3 and H3K27me3 were largely unchanged from testicular sperm to epididymal sperm in wild-type males, demonstrating that the epididymal pattern of sperm epigenome is already established in testicular sperm.

In *Scml2*-KO males, distribution patterns of these marks overall resemble that of wild-type sperm. However, the intensity of these marks changed in *Scml2*-KO males. On Cluster 2 peaks, H3K4me3 was increased, but H3K27me3 was attenuated in *Scml2*-KO testicular sperm; H3K27me3 was further attenuated in *Scml2*-KO epididymal sperm (Figure 2B). Cluster 2 peaks are associated with developmental regulator genes such as neuron, gland, and heart development (Figure 2C). Consistent with these results, our promoter-specific analysis further confirmed that epigenetic states of developmental regulator genes were altered in *Scml2*-KO testicular and epididymal sperm (Supplementary Figure S3). We further found an extensive reduction of H3K27me3 in *Scml2*-KO epididymal sperm on Cluster 4 peaks (Figure 2D), which is associated with other somatic cellular processes (Figure 2E). Taken together, we conclude that SCML2 is required for the establishment of the proper epigenome in testicular sperm and epididymal sperm and that *Scml2*-KO epididymal sperm has more pronounced defects compared to *Scml2*-KO testicular sperm.

### Paternal *Scml2* deficiency leads to altered sperm RNA content in the next generation

**Figure 3.**
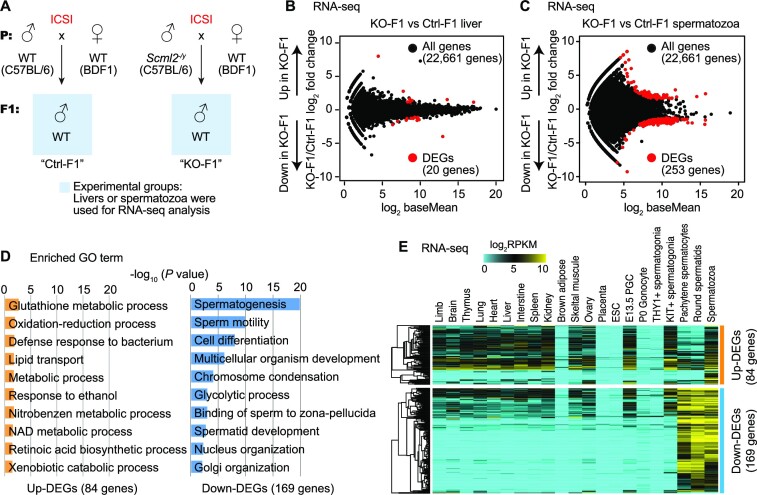
Paternal *Scml2* deficiency leads to altered sperm RNA content in the next generation. (**A**) Schematic to obtain experimental samples from Ctrl and *Scml2*-KO ICSI-derived offspring (F1). (**B, C**) RNA-seq analysis of livers and spermatozoa from F1 mice. Differentially expressed genes (DEGs) were defined with stringent criteria (fold change > |2|, *P*_adj_ < 0.01). In liver RNA-seq, only a few DEGs were detected between Ctrl and KO-derived F1 offsprings. (**D**) Bar charts showing enriched GO terms for the upregulated (up-) and downregulated (down-) DEGs between Ctrl and KO-derived F1 spermatozoa. (**E**) Heatmaps depicting expression pattern (log_2_ RPKM) of up-DEGs (top) and down-DEGs (bottom) in *Scml2*-KO F1 spermatozoa among several somatic tissues and male germline cells. ESC: embryonic stem cells. PGC: primordial germ cells.

To determine whether SCML2 mediates epigenetic inheritance between generations (i.e. intergenerational inheritance), we sought to determine changes in gene expression in *Scml2*-KO-derived F1 (KO-F1) males obtained from ICSI with testicular sperm. To this end, we performed RNA-seq analyses of livers and spermatozoa in KO-F1 males in comparison with the control F1 (Ctrl-F1) males that derived from wild type (*Scml2*^+/y^) males using ICSI (Figure 3A, Supplementary Figure S4A); these comparisons were made between wild-type mice generated from ICSI to reflect true epigenetic differences. We selected the liver because the liver is a representative organ that shows the effect of paternal epigenetic inheritance in the case of low-protein diet-fed fathers (3). However, we detected only a few differentially expressed dysregulated genes between KO-F1 and Ctrl-F1 liver (Figure 3B, Supplementary Dataset S1), suggesting that paternal *Scml2* deficiency has a minor impact on gene expression in the F1 liver. We next analyzed F1 spermatozoa isolated from the cauda epididymis. Because SCML2 is a regulator of gene expression in germ cells (37), we suspect that SCML2-dependent paternal epigenetic states may persist on sperm chromatin to regulate gene expression in the germline of the next generation. We first confirmed that our sperm RNA-seq did not exhibit expression of somatic markers in the epididymis (58) (Supplementary Figure S4B). We found that 253 genes were defined as differentially expressed genes (DEGs) between KO-F1 and Ctrl-F1 spermatozoa (Figure 3C, Supplementary Dataset S1). Because active transcription is ceased in spermatozoa, these results suggest that SCML2-dependent paternal epigenetic states alter gene expression in spermiogenesis in KO-F1 males, leading to altered RNA content in KO-F1 spermatozoa. Despite the altered RNA content, KO-F1 males were fertile and produced offspring in natural mating (F2 analysis is described in a later section).

Among 253 DEGs in KO-F1 spermatozoa, 84 genes were upregulated in KO-F1 spermatozoa, while 169 genes were downregulated. Gene ontology (GO) enrichment analysis revealed that these upregulated genes are enriched with gene functions in various biological processes such as glutathione metabolic process and oxidation-reduction process (Figure 3D). By contrast, down-regulated genes were highly enriched with gene functions in spermatogenesis and sperm motility (Figure 3D). Further, we examined expression of these DEGs in many other tissues and different stages of isolated germ cells and confirmed late-spermatogenesis specific expression of 169 downregulated genes (down-DEGs) in KO-F1 sperm; these downregulated genes are highly expressed in meiotic pachytene spermatocytes (PS), round spermatids (RS), and spermatozoa (Figure 3E). On the other hand, many of the 84 upregulated genes (up-DEGs) in KO-F1 spermatozoa are highly expressed in various somatic tissues and some stages of differentiated germ cells (such as KIT^+^ spermatogonia and spermatozoa), though these 84 up-DEGs are not highly expressed in undifferentiated cells (such as ESC and THY1^+^ undifferentiated spermatogonia) and in later spermatogenesis (PS and RS). Consistent with the late spermatogenesis functions of 169 down-DEGs in KO-F1 sperm, these genes tend not to be highly conserved in mammals. Among them, 20 rodent-specific genes are observed, which are in line with the rapid evolution of late spermatogenesis genes (Supplementary Figure S5). Together, we conclude that paternal *Scml2* deficiency leads to altered sperm RNA content in the next generation in two major groups: downregulation of late spermatogenesis genes and upregulation of differentiation-related genes. These results indicate that SCML2 mediates intergenerational inheritance of epigenetic information.

### Histone retention at *Scml2* KO-F1 dysregulated gene loci in wild-type spermatozoa

**Figure 4.**
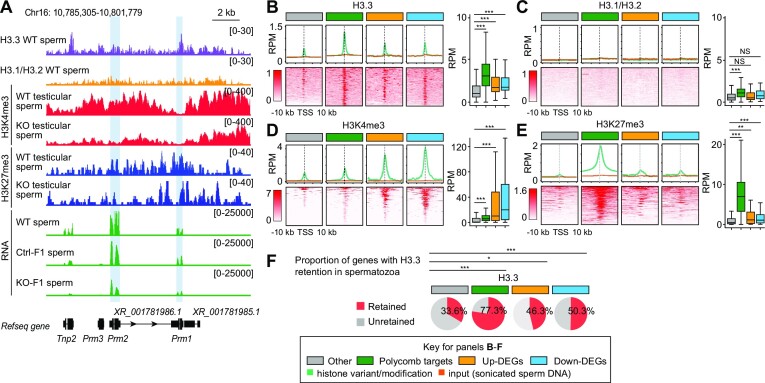
Dysregulated gene loci in *Scml2*-KO ICSI-derived sperm showed histone retention. (**A**) Representative genome browser track view of each histone variant/modification ChIP-seq reads from WT epidydimal sperm (H3.3 and H3.1/3.2, adapted from (15)), WT and *Scml2*-KO testicular sperm (H3K4me2 and H3K27me3, generated in this study), and RNA-seq reads from WT (adapted from (70)), Ctrl- and KO-ICSI derived F1 sperm (generated in this study). *Tnp2*, *Prm1*, *Prm2* and *Prm3* genes were extracted as down-DEGs in KO-F1 spermatozoa. Data ranges are shown in brackets. The *Prm1* and *Prm2* gene loci were highlighted with blue. (B-E) Average tag density plots and heatmaps around TSS ±10 kb, as well as whisker plots of enrichment around TSSs ±1 kb of H3.3, H3.1/H3.2, H3K4me3 and H3K27me3 in wild-type sperm ChIP-seq reads on gene groups shown in upper color insets. *P < 0.05; **P < 0.01; ***P < 0.001; NS, not significant; Wilcoxon rank-sum test. Whisker plots: Central bars represent medians, the boxes encompass 50% of the data points, and the whiskers indicate 90% of the data points. (**F**) The proportion of genes with H3.3 retention in spermatozoa on gene groups shown in upper color insets. Genes with >1.5-fold enrichment compared to inputs were defined as genes with H3.3 retention. *P < 0.05; ***P < 0.001. Chi-square test with Yates correction.

To delineate mechanisms associated with SCML2-mediated paternal epigenetic inheritance, we examined the chromatin status of the DEGs in wild-type spermatozoa. A representative locus of down-DEGs of KO-F1 spermatozoa comprises the Protamine gene cluster, which contains three protamine genes (*Prm1*, *Prm2* and *Prm3*) and a transition nuclear protein gene (*Tnp2*) on chromosome 16, all of which are coregulated as a cluster and highly expressed in spermiogenesis (59). In KO-F1 sperm, these genes were significantly downregulated (Figure 4A, Supplementary Dataset S1), raising the possibility that SCML2 regulates the Protamine gene cluster. We reanalyzed the published ChIP-seq data set (15) and found that *Prm1*, *Prm2*, *Prm3* and *Tnp2* loci are enriched with histone variants H3.3, which is a major component of retained histones in spermatozoa (15). These gene loci were associated with H3.3 in spermatozoa (15). Further, the Protamine gene cluster is broadly enriched with H3K27me3, a PRC2 mediated repressive mark regulated by SCML2 in the germline (29). Indeed, H3K27me3 was attenuated at the *Prm1* and *Prm2* gene loci in *Scml2*-KO testicular sperm (highlighted with blue bars in Figure 4A). Thus, histone retention at the Protamine gene cluster and the presence of H3K27me3 in spermatozoa is a possible mechanism by which SCML2 mediates paternal epigenetic inheritance.

We further investigated the genome-wide features of these DEGs in wild-type spermatozoa. We compared average tag density of H3.3, canonical histone H3 (H3.1/H3.2), H3K4me3 and H3K27me3 in wild-type spermatozoa over the four groups of genes: up- and down-DEGs in KO-F1 spermatozoa, canonical Polycomb target genes (3599 genes) identified in ESC (60), and other genes in the genome. Compared to other genes, up- and down-DEGs in KO-F1 spermatozoa are enriched with H3.3 (Figure 4B, while canonical H3.1/H3.2 did not show notable enrichment on these genes (Figure 4C). Consistent with the example of the Protamine gene cluster, up- and down-DEGs in KO-F1 spermatozoa are enriched with H3K4me3 to a higher degree than Polycomb target genes (Figure 4D). H3K27me3 was also significantly enriched on up- and down-DEGs in KO-F1, but to a lesser extent compared to canonical Polycomb target genes (Figure 4E). Further, proportions of genes with histone retention were estimated based on H3.3 enrichment: 46.3% and 50.3% of up- and down-DEGs show > 1.5-fold enrichment of H3.3 (normalized to input read) in spermatozoa (Figure 4F). These analyses demonstrate that, in wild-type spermatozoa, histone retention takes place on dysregulated gene loci detected in KO-F1, raising the possibility that SCML2 mediates intergenerational epigenetic inheritance through sperm chromatin.

### SCML2 establishes H3K27me3 on *Scml2*-KO-F1 dysregulated gene loci in late spermatogenesis

**Figure 5.**
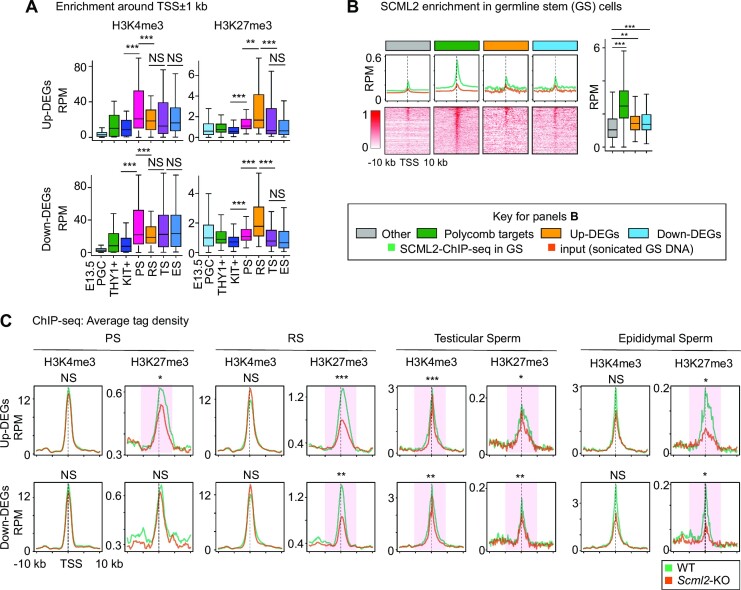
SCML2-dependent establishment of H3K27me3 on the *Scml2*-KO-F1 dysregulated gene loci. (**A**) Whisker plots of enrichment around TSSs ±1 kb of H3K4me3 and H3K27me3 ChIP-seq read in each stage of male germ cells. **P < 0.01; ***P < 0.001; NS, not significant; Wilcoxon rank-sum test. Central bars represent medians, the boxes encompass 50% of the data points, and the whiskers indicate 90% of the data points. E13.5 PGC: E13.5 male primordial germ cells, THY1+:THY1+ undifferentiated spermatogonia, KIT+:KIT1+ differentiating spermatogonia. PS: pachytene spermatocytes, RS: Round spermatids, TS: Testicular sperm, ES: epididymal sperm. (**B**) Average tag density plots and heatmaps around TSS ±10 kb, as well as whisker plots of enrichment around TSSs ±1 kb of SCML2 ChIP-seq reads in germline stem (GS) cells on gene groups shown in upper color insets. **P < 0.01; ***P < 0.001; Wilcoxon rank-sum test. Whisker plots: Central bars represent medians, the boxes encompass 50% of the data points, and the whiskers indicate 90% of the data points. (**C**) Average tag density plots of H3K4me3 and H3K27me3 ChIP-seq reads around TSS (±10 kb) of up- and down-DEGs in PS, RS, testicular sperm and epididymal sperm from wild-type and *Scml2*-KO males. **P < 0.01; ***P < 0.001; NS, not significant; Wilcoxon rank-sum test with Bonferroni correction was performed around TSS (±5 kb) (shown with pink areas).

We next examined how the *Scml2*-KO-F1 dysregulated gene loci are modified in the male germline. Of note, H3K4me3 and H3K27me3 are present on up- and down-DEGs loci in E13.5 primordial germ cells, which undergo extensive epigenetic reprogramming (reanalysis from (61): Figure 5A and Supplementary Figure S6). At these gene loci, H3K4me3 and H3K27me3 are present in THY1^+^ undifferentiated spermatogonia and KIT^+^ differentiating spermatogonia (reanalysis from (29)). Subsequently, H3K4me3 increases in PS in meiosis, followed by an increase of H3K27me3 in RS in the postmeiotic stage (reanalysis from (29): Figure 5A). After RS, the intensity of H3K27me3 decreases in testicular sperm but remains present in epididymal sperm, while H3K4me3 remains high after RS (original data in this study: Figure 5A). The late establishment of H3K27me3 after H3K4me3 appears to be explained by the function of SCML2 as the regulator of bivalent chromatin; SCML2 binds to hypomethylated promoter marked with H3K4me3 and induces H3K27me3 to establish bivalent chromatin status (29). This notion is further supported by the analysis of SCML2-binding sites. We reanalyzed the SCML2 binding sites detected in cultured germline stem cells (37) and found that SCML2 directly binds to promoters of both up- and down-DEGs, and the intensity of SCML2 signals is high on these DEG loci compared to other gene loci (Figure 5B). We next examined the function of SCML2 using the ChIP-seq data from *Scml2*-KO PS and RS (29) and native ChIP-seq data generated in this study. Although statistically significant changes were not observed on *Scml2*-KO PS for H3K4me3 and H3K27me3 enrichment on down-DEGs, H3K27me3 enrichment on up-DEGs in PS, RS, testicular sperm, and epididymal sperm was decreased in *Scml2*-KO (Figure 5C). H3K27me3 enrichment on down-DEGs in RS, testicular sperm and epididymal sperm was also decreased in *Scml2*-KO (Figure 5C). These results suggest that the SCML2-dependent establishment of H3K27me3 in the male germline is correlated with SCML2 mediated paternal epigenetic inheritance.

### Intergenerational effects were largely corrected in F2 mice sired from *Scml2-*KO males

**Figure 6.**
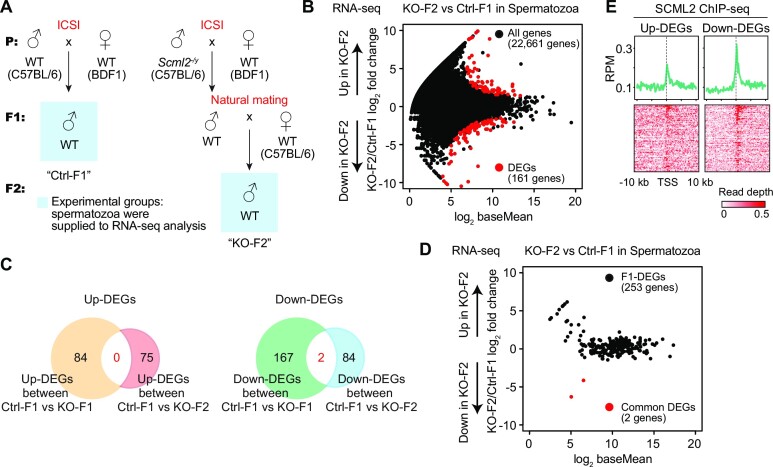
Instability of epigenetic information in the F2 mice sired from *Scml2*-KO males. (**A**) Strategy for generation and mating of Ctrl and *Scml2*-KO offsprings. For RNA-seq analysis, Ctrl-F1 and *Scml2*-KO-F2 spermatozoa were compared. (**B**) The mRNA-seq analysis in sperm samples from Ctrl-F1 and *Scml2*-KO-F2. 161 genes were significantly dysregulated (fold change > |2|, *P*_adj_ < 0.01) in spermatozoa of KO-F2, shown in red circles. (**C**) Venn diagrams showing the overlap of DEGs between *Scml2*-KO-F1 and F2 sperms. (**D**) The mRNA-seq analysis in sperm samples from Ctrl-F1 and *Scml2*-KO F2. MA plot showing expression patterns of 253 dysregulated genes are detected in Figure 3 between Ctrl-F1 and *Scml2*-KO F2. The remaining DEGs in spermatozoa of KO-F2 are shown in red circles. (**E**) Average tag density plots and heatmaps of GS SCML2 ChIP-seq signals around TSSs of DEGs in *Scml2*-KO-F2 spermatozoa (±10 kb).

Next, we sought to address whether SCML2-mediated epigenetic states persist across generations (i.e. transgenerational inheritance). To this end, we obtained F2 males from the KO-F1 males sired from *Scml2*-KO males (KO-F2; Figure 6A); the KO-F1 males were fully fertile, despite the dysregulation of gene expression in spermatozoa (Figure 3). To test the germline gene expression dysregulated in KO-F2 spermatozoa, we performed RNA-seq of KO-F2 spermatozoa and compared it with F1 control spermatozoa (Supplementary Figure S7A, B). We detected 161 dysregulated genes *Scml2*-KO F2 spermatozoa; 75 genes were up-DEGs and 86 genes were down-DEGs in KO-F2 spermatozoa (Figure 6B, Supplementary Dataset S2). Of note, only two genes were overlapped between down-DEGs in KO-F1 spermatozoa and KO-F2 spermatozoa, while there was no overlap between up-DEGs in KO-F1 spermatozoa and KO-F2 spermatozoa (Figure 6C, D). This suggests that, in KO-F2, there are no consistent set of DEGs that correlate with the effects observed in the first generation (KO-F1) and that epigenetic abnormally detected in KO-F1 spermatozoa were largely corrected in the F2 generation. Consistent with this idea, GO enrichment analysis revealed distinct classes of genes are dysregulated in between KO-F1 spermatozoa and KO-F2 spermatozoa, and we did not observe an enrichment of spermatogenesis-related genes in down-DEGs of KO-F2 spermatozoa (Supplementary Figure S7C). However, SCML2 peaks were detected at the promoters of up- and down-DEGs of KO-F2 spermatozoa in cultured germline stem cells (Figure 6E). In our experiments, we used a consistent genetic background, and we confirmed that biological replicates for our data sets are similar to each other (Supplementary Figure S7B), excluding the possibility that the KO-F2 results were caused by differences in genetic background or individual differences. An alternative possibility could be instability of epigenetic information in the F2 mice sired from *Scml2-*KO males, while similar patterns of gene dysregulation were not observed between KO-F1 spermatozoa and KO-F2 spermatozoa. Therefore, intergenerational effects were largely corrected in F2 mice sired from *Scml2-*KO males. It is still possible that SCML2 may impact transgenerational inheritance of epigenetic information. However, the instability is unlikely to be explained by stable transmission of epigenetic states at specific target loci across generations.

### Dysregulation of gene expression in preimplantation embryos derived from *Scml2*-KO testicular sperm

**Figure 7.**
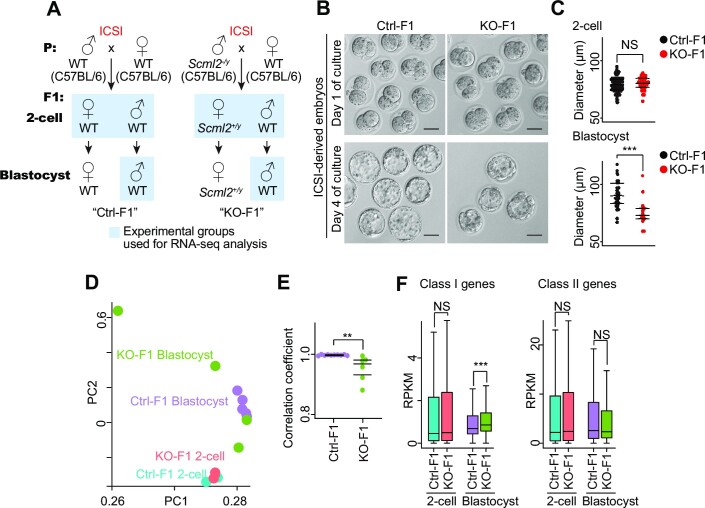
Dysregulation of gene expression in preimplantation embryos derived from *Scml2*-KO testicular sperm. (**A**) Strategy for generation of Ctrl-F1 and *Scml2*-KO-F1 preimplantation embryos by ICSI using testicular sperm. (**B**) Representative phase-contrast images of F1 preimplantation embryos at the two-cell (Day 1) and blastocyst stages (Day 4), generated by ICSI with testicular sperm from Ctrl and *Scml2*-KO males, followed by *in vitro* culture. Scale bars, 50 μm. (**C**) Dot plots showing diameters for F1 embryos at the two-cell and blastocyst stages derived from Ctrl and *Scml2*-KO testicular sperm. Central bars represent medians; the top and bottom lines encompass 50% of the data points. NS, not significant; ***P< 0.001; two-tailed unpaired *t*-test. (**D**) Bidimensional principal-component (PC) analysis of gene expression profiles in ICSI-derived Ctrl- and *Scml2*-KO-F1 embryos at the two-cell and blastocyst stages. (**E**) Dot plots showing the Pearson correlation coefficient values for each pair of single XY blastocyst transcriptomes in Ctrl- and *Scml2*-KO-F1 groups. Central bars represent medians; the top and bottom lines encompass 50% of the data points. **P< 0.01; two-tailed unpaired *t*-test. (**F**) Box-and-whisker plots showing the RPKM values for *Scml2*-target genes (as defined in (29); Class I, developmental regulator genes; Class II, somatic genes) in ICSI-derived Ctrl- and *Scml2*-KO-F1 embryos at two-cell and blastocyst stages. Central bars represent medians, boxes encompass 50% of the data points, and the whiskers indicate 90% of the data points. NS, not significant; ***P< 0.001; Wilcoxon rank-sum test.

Finally, we sought to determine how paternal chromatin defects in *Scml2*-KO testicular sperm impact the next generation after fertilization. To this end, we performed ICSI experiments using *Scml2*-KO and wild-type control testicular sperm and examined gene expression in preimplantation embryos derived from them. To precisely evaluate the gene expression, we used oocytes from C57BL/6 female mice, which have the same genetic background as the *Scml2*-KO male mice (Figure 7A). After ICSI, KO-derived (KO-F1) and wild-type control-derived (Ctrl-F1) embryos were cultured for a day to obtain two-cell embryos and for 4 days to obtain blastocysts (Figure 7B). KO-F1 embryos developed into blastocysts, which showed abnormal morphology (Figure 7B) and a smaller diameter (Figure 7C) with slightly less frequency (Supplementary Table S2) compared to Ctrl-F1 blastocysts. We performed RNA-seq analyses of pooled two-cell embryos, in which KO-F1 embryos consist of *Scml2*^+/−^ females and wild-type males, and further performed RNA-seq analyses of each single male blastocysts (four KO-F1 blastocysts and five Ctrl-F1 blastocysts: Figure 7A, Supplementary Figure S8A, B). Because *Scml2* is the X-linked gene, all male embryos derived from *Scml2*-KO sperm are wild-type. Thus, we were able to evaluate true epigenetic effects by analyzing single male blastocysts with the same wild-type genotype between KO-F1 blastocysts and Ctrl-F1 blastocysts. Although gene expression profiles of pooled two cell samples showed largely consistent between KO-F1 and Ctrl-F1 and between biological replicates, individual KO-F1 blastocysts showed variable gene expression compared to Ctrl-F1 blastocysts (Figure 7D, E). These results demonstrated that the epigenetic abnormality of *Scml2*-KO testicular sperm was associated with abnormal gene expression in genetically wild-type blastocysts (KO-F1).

In these RNA-seq data sets, a small number of DEGs were detected between KO-F1 and Ctrl-F1 two-cell embryos (Supplementary Figure S8C, Supplementary Dataset S3). On the other hand, 535 upregulated genes and 312 downregulated genes were detected when we compared KO-F1 and Ctrl-F1 blastocysts, while a few repetitive elements were found to be upregulated in KO-F1 blastocysts (Supplementary Figure S8C, Supplementary Dataset S3). We previously showed that SCML2 regulates H3K27me3 on two distinct classes of bivalent genes; a class includes developmental regulator genes that are not active in the germline (Class I) and somatic/mitotic genes that are suppressed in late spermatogenesis (Class II) (29). Based on these findings, we next examined whether these target genes are dysregulated in the embryos. Notably, the Class I genes were specifically upregulated in KO-F1 blastocysts (Figure 7F). A GO term analysis revealed that the upregulated genes in KO-F1 blastocysts are enriched with genes related to developmental processes, including the GO terms ‘regulation of neuron differentiation’, ‘cardiocyte differentiation’, and ‘cardiac cell development’ (Supplementary Figure S8D). Further, these DEGs in KO-F1 male blastocysts were the SCML2 target genes in the male germline; SCML2 accumulated on DEGs in germline stem cells, and H3K27me3 accumulation on these genes was SCML2-dependent in representative stages of the male germline (Supplementary Figure S9). Therefore, these results suggest that paternal epigenetic defects in *Scml2*-KO sperm were associated with the misregulation of developmental regulator genes in KO-F1 blastocysts.

## DISCUSSION

In this study, we present functional evidence that the classic epigenetic regulator Polycomb mediates paternal epigenetic inheritance through sperm chromatin. We rescued the infertility of *Scml2*-KO males and demonstrated that the *Scml2*-KO mouse line serves as a novel model of epigenetic inheritance, particularly that of paternal epigenetic states. We further provide novel epigenomic resources for testicular sperm. We show that epididymal pattern of the sperm epigenome is already established in testicular sperm. Although we were not able to determine an exact reason why testicular sperm of *Scml2*-KO males worked with ICSI, possible reasons include deterioration of chromatin states or genome integrity from testicular sperm to epididymal sperm of *Scml2*-KO males. In line with this possibility, H3K27me3 was further attenuated from testicular sperm to epididymal sperm of *Scml2*-KO males at specific loci (Figure 2B). Another alternative possibility could be structural defects of *Scml2*-KO epididymal sperm. *Scml2*-KO epididymal sperm have midpieces that are tightly associated with nuclei; we were not able to dissociate their midpieces from sperm nuclei, which might have prevented ICSI from working in our previous study (29). In this study, we used nuclei of cryopreserved *Scml2*-KO testicular sperm for ICSI. These sperm nuclei were detached from midpieces after cryopreservation and prior to ICSI.

**Figure 8.**
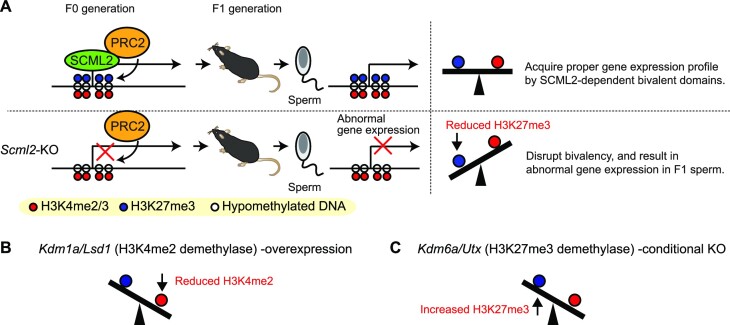
Model: SCML2-mediated epigenetic inheritance and its relevance to other mouse models. (**A**) SCML2 interacts with PRC2 and facilitates H3K27me3 on the hypomethylated promoters of target genes in late spermatogenesis. SCML2-mediated establishment of bivalent chromatin defines epigenetic states of germline lines in the F1 generation. (**B**) *Lsd1* (H3K4me2 demethylase) -overexpression leads to decreased H3K4me2. Siklenka *et al.* and Lismer *et al.* (30,31). (**C**) *Kdm6a*/*Utx* (H3K4me2 demethylase) -conditional KO leads to increased H3K27me3. Lesch *et al.* (33).

In late spermatogenesis, SCML2 binds to hypomethylated promoters of target genes where H3K4me3 is enriched and interacts with PRC2 to establish H3K27me3 on its target gene loci, leading to the establishment of bivalent genomic domains (29) (Figure 8A). SCML2 interacts with both PRC1 and PRC2 (29,37). However, PRC1-mediated H2AK119ub was not observed in normal late spermatogenesis (37). On the other hand, PRC2-mediated H3K27me3 is extensively established during late spermatogenesis in an SCML2-dependent manner (29). Therefore, it is reasonable to postulate that the *Scml2*-KO phenotypes emanate from SCML2-dependent regulation of PRC2 in late spermatogenesis. We found that histone H3.3 was retained, and H3K4me3 and H3K27me3 were present in spermatozoa, whereas H3.1/2 levels were very low on the promoters of dysregulated gene loci of our *Scml2*-KO-F1 model. These findings support the possibility that H3.3 in sperm may be modified with trimethylation at K4 and K27 sites and is responsible for the inheritance of paternal epigenetic states. In spermatogenesis, testis-specific histone variant H3T is a major isoform of histone H3 and is considered to be replaced by H3.3 in late spermatogenesis (62). Thus, SCML2 mediated trimethylation at K27 may occur on H3.3 after replacing H3T at the promoter regions of target loci, and the epigenetic states may persist into the next generation to regulate gene expression in late spermatogenesis of F1.

A recent study showed that multiple histone modifications undergo changes during sperm maturation in the epididymis, some of which occurred on H3.3 (63). In accord with this observation, we show that H3K27me3 is further attenuated in epididymal sperm of *Scml2*-KO compared to testicular sperm (Figure 2B), raising the possibility that alterations of H3.3 modifications during sperm maturation are affected in *Scml2*-KO sperm. Further, SCML2 is a regulator of germline gene expression (37); thereby, it is possible that SCML2 establishes chromatin states that modulate germline gene expression in the next generation. This possibility is in line with the abnormal gene expression in *Scml2*-KO-F1 blastocysts. We speculate that SCML2 deletion in the paternal germline causes epigenetic instability on target loci, which are again regulated in the germline of the offsprings. To test this possibility in future studies, it would be important to determine the epigenetic states of *Scml2*-KO-F1. In addition, there remains an important question as to the impact of paternal SCML2 loss on the soma of the offsprings, although we found it has a minor impact on the liver.

Because active H3K4me2/3 and silent H3K27me3 counteract each other at bivalent chromatin in the germline (29), it is possible that the balanced establishment of bivalent domains may be required for the proper inheritance of epigenetic states for gene regulation in the germline. In *Scml2*-KO-F1 blastocysts, developmental regulator genes that carry bivalent chromatin were dysregulated, although such extensive dysregulation could lead to embryonic death and the survival of relatively normal embryos after implantation. Accordingly, a possible explanation for the F1 germline phenotype is that the attenuation of H3K27me3 may result in abnormal gene expression of F1 spermiogenesis (Figure 8A). Although bivalent chromatin is a persistent feature in the germline, a major unsolved mystery is how epigenetic states are inherited and maintained throughout the germline after fertilization. While paternal H3.3 on promoters disappears after fertilization (64) and H3K27me3 is reprogrammed in preimplantation development (60), a recent study showed that paternal H3K4me3 persists through preimplantation development (30). Thus, it is possible that the paternal epigenetic states, reflecting the memory of paternal H3K27me3, escape epigenetic reprogramming and persist at the promoter regions of the target genes throughout germ cell development.

In support of this idea, we found that H3K4me3 and H3K27me3 persist on the target genes, albeit at low levels, in E13.5 PGC (Figure 5A and Supplementary Figure S6), where extensive epigenetic reprogramming takes place. Because SCML2 is required for H3K27me3 establishment in the male germline, SCML2 deletion in the paternal germline may perturb epigenetic states in offspring. This notion is in part supported by the analyses of *Scml2*-KO-F1 blastocysts. Our recent study compared embryos derived from ROSI and ICSI using epididymal sperm, and we found that paternal H3K27me3 is linked to the gene expression change in early embryos (65). This study further supports our conclusion that paternal H3K27me3 is involved in intergenerational epigenetic inheritance. On the other hand, the KO-F1 and KO-F2 showed apparently distinct patterns of gene expression, suggesting that epigenetic states may be largely reprogrammed in each generation, though a sort of epigenetic instability may be transmitted across generations.

Of note, our new model of epigenetic inheritance may have some relevance with the previously established model of paternal epigenetic inheritance through sperm chromatin: the *Kdm1a* overexpression model, which reduces H3K4me2 in the germline (30,31) (Figure 8B), and the *Kdm6a* conditional deletion model, which potentially increases H3K27me3 in the germline (33) (Figure 8C). The precise mechanisms of action underlying these mouse models remain elusive; yet, together with our study, it is tempting to speculate that the balance between Polycomb-mediated H3K27me3 and H3K4me2/3 at regulatory elements may be responsible for the establishment of proper gene expression in the next generation.

At the end of this study, we note that there remains a major unsolved question as to how chromatin states can be bookmarked, escape epigenetic reprogramming, and persist at the promoter regions of the target throughout the germline. A possibility is that the abnormal H3K27me3/H3K4me3 could affect DNA methylation or transcription factor binding to gene regulatory elements in the germline (66). Another possibility could be that these sites may escape epigenetic reprogramming, as some TE-derived locus escape epigenetic reprogramming in PGC (67). The notion of chromatin-based persistent epigenetic abnormality is in line with the other emerging cases of epigenetic inheritance (68,69). Further, the severe blastocyst phenotype of *Scml2*-KO-F1 blastocysts suggests that there could be a broad impact on F1 soma via intergenerational inheritance. However, the severe blastocyst phenotype could be enhanced due to in vitro culture because we did not observe a significant difference in reproduction outcomes in two-cell transferred embryos (Supplementary Table S1). Although much remains unknown in paternal epigenetic inheritance, our development of a new tractable mouse model will open up new avenues for future investigations.

## Supplementary Material

gkad479_Supplemental_Files

## Data Availability

NGS datasets used in this study are publicly available and referenced within the article. All 59 datasets from other resources are listed and referenced in Supplementary Dataset S4. All the RNA-seq and native ChIP-seq data generated in this study are deposited to the Gene Expression Omnibus (GEO) under accession code GSE183994.
