# Supplementary material for: Polycomb protein SCML2 mediates paternal epigenetic inheritance through sperm chromatin
Source: Nucleic Acids Res. 2023 Jun 7;51(13):6668–83. doi: 10.1093/nar/gkad479 (PMC10359620; doi:10.1093/nar/gkad479)
Supplement: gkad479_Supplemental_Files [file gkad479_supplemental_files.zip › 060123SupInformation.pdf]

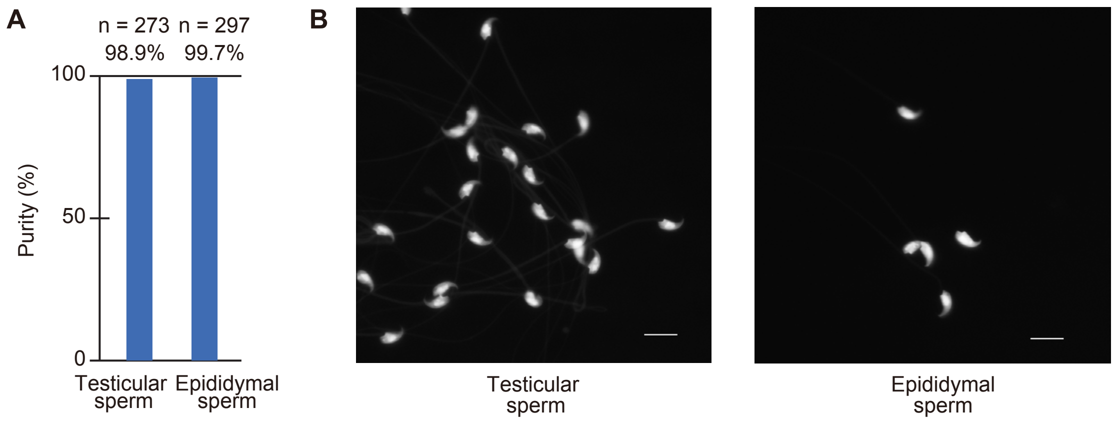

Supplementary Figure S1. Validation of isolated testicular sperm and cauda epididymal sperm.
(**A**) Purity of each sperm sample collected from the testis (testicular sperm) and cauda epididymis (epididymal sperm). Validation was performed using 2 independent wild-type male mice at 2 months old. Purity was determined by the proportion of sperm in all DAPI-positive cells (Numbers of counted DAPI-positive cells were shown).
(**B**) Representative DAPI-stained images of each fraction for testicular sperm and epididymal sperm. Scale bars, 10µm.

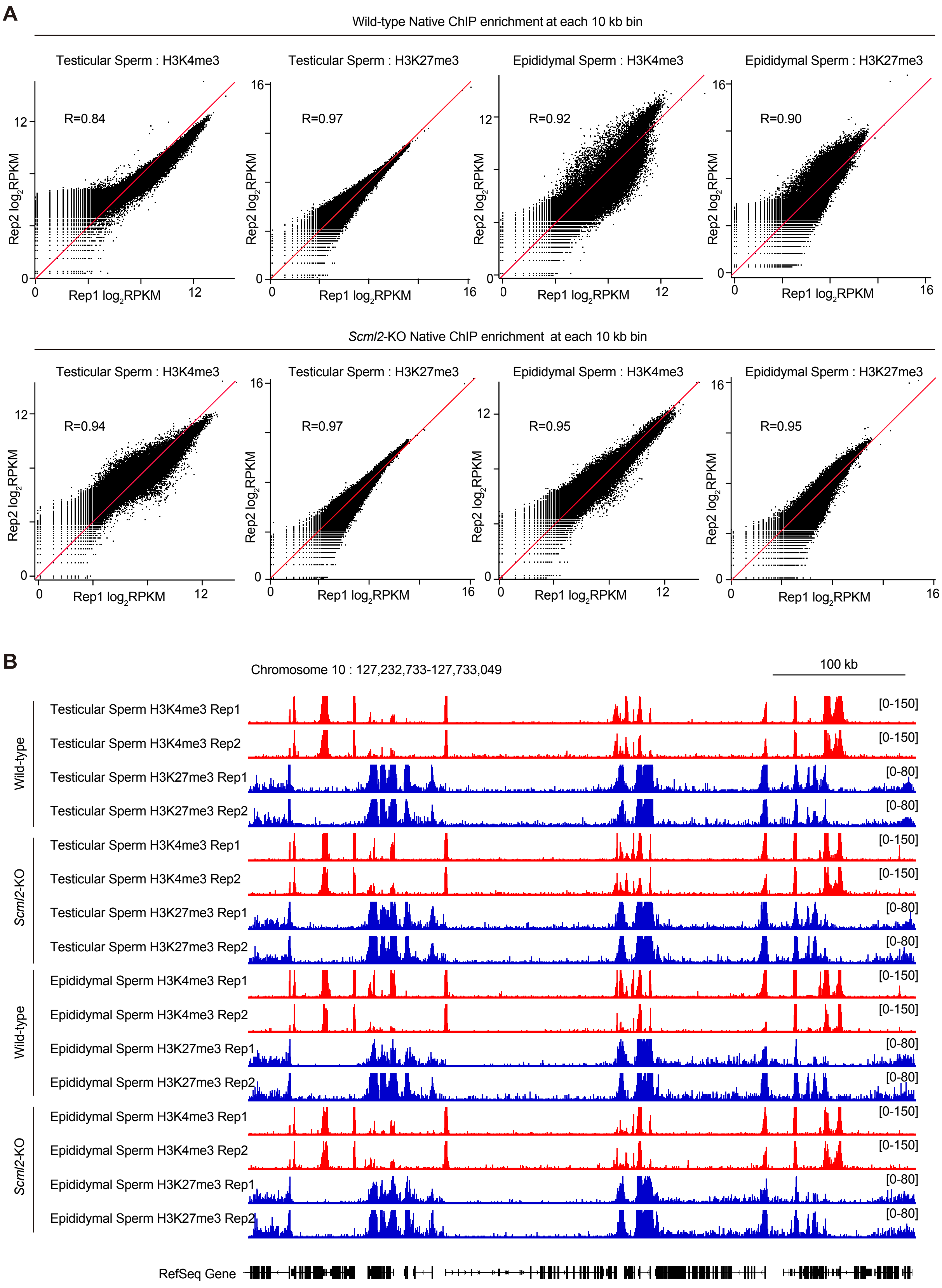

Supplementary Figure S2. Validation of biological replicates in the ChIP-seq experiments.
(**A**) Scatter plots show the correlation between biological replicates of native ChIP-seq enrichments at each 10kb bin. ChIP-seq enrichment levels are shown in $\log_2$ RPKM values. Pearson correlation values (R) are shown.
(**B**) Track views of ChIP-seq enrichments with biological replicates.

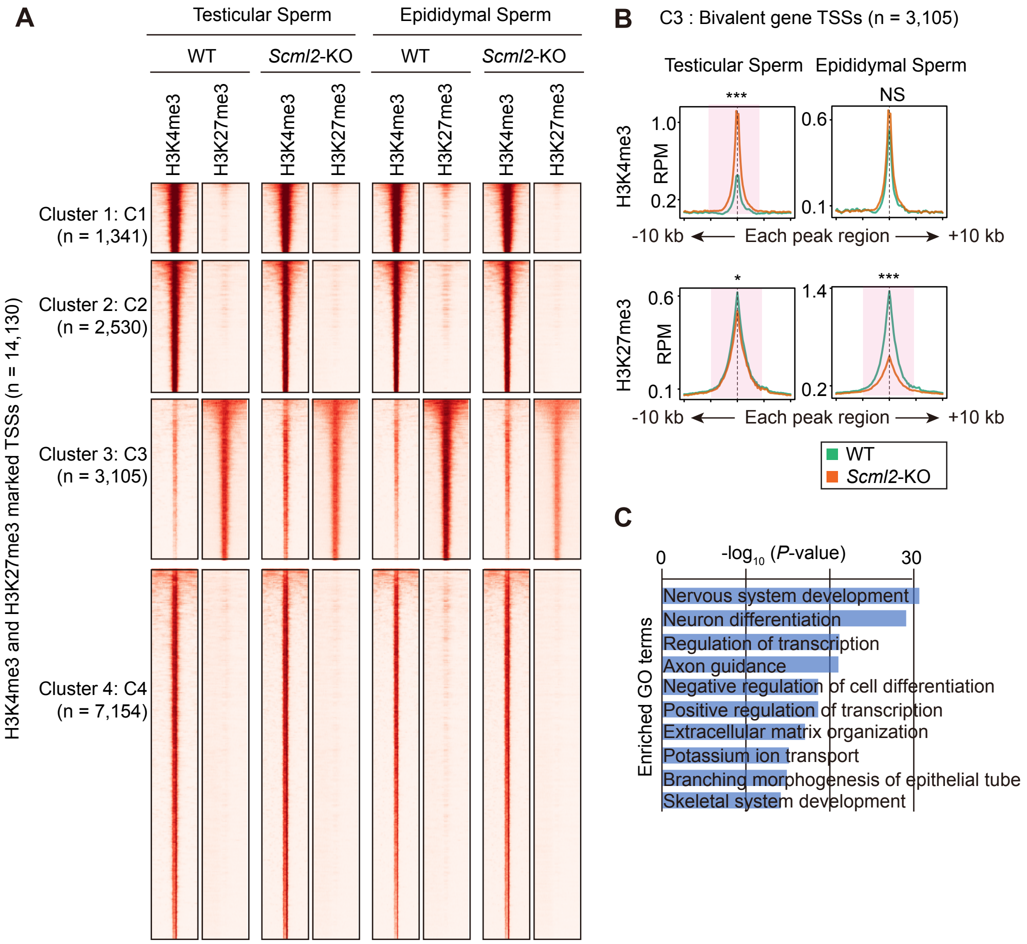

Supplementary Figure S3. H3K27me3 and H3K4me3 distributions on promoters (transcription start sites: TSSs) in testicular sperm and epididymal sperm.

(**A**) k-means clustered heatmap of H3K4me3 and H3K27me3 marked TSSs in wild-type and *Scml2*-KO testicular sperm and epididymal sperm.

(**B**) Average tag density plots around TSSs ±10 kb for Cluster 3: C3. *P < 0.05; ***P < 0.001; NS, not significant; Wilcoxon rank-sum test with Bonferroni correction was performed around TSS (±5 kb) (shown with pink areas).

(**C**) Bar charts showing enriched GO terms for genes in Cluster 3.

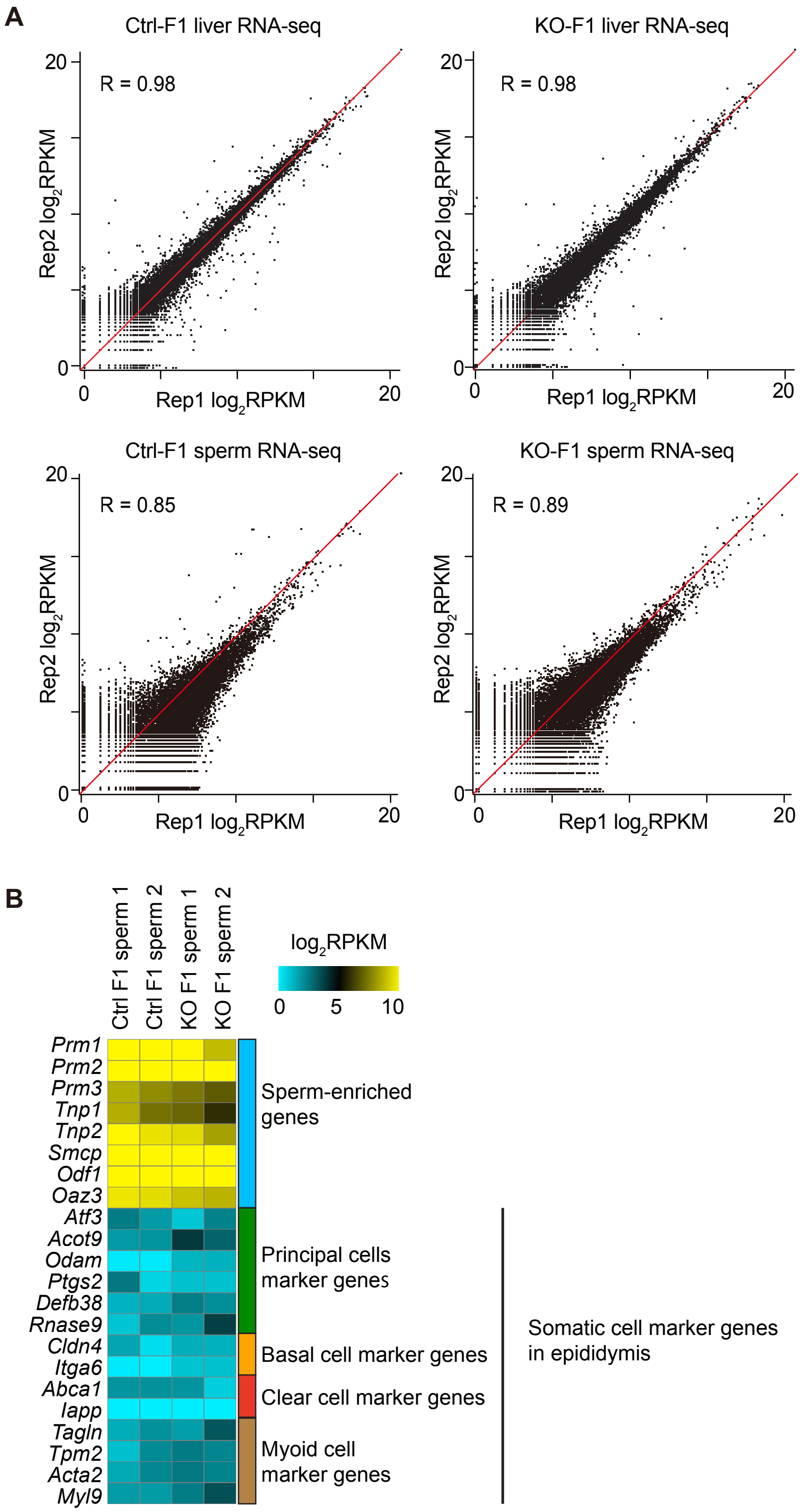

Supplementary Figure S4. RNA-seq analyses of liver and sperm from *Scml2*-KO F1 and their controls.
(**A**) Scatter plots show the correlation between biological replicates of RNA-seq reads for each gene. Expression levels for each gene are shown in $\log_2$ RPKM values. Pearson correlation values (R) are shown.
(**B**) The expression level of sperm enriched genes and epididymal somatic cell marker genes in the RNA-seq of F1 epididymal sperm. Heatmaps depicting expression pattern ($\log_2$ RPKM) of sperm enriched genes and epididymal somatic cell marker genes (reported in Rinaldi *et al., eLife* 2020) in epididymal sperm of F1 offspring derived from wild-type and *Scml2*-KO males.

Sequence identity

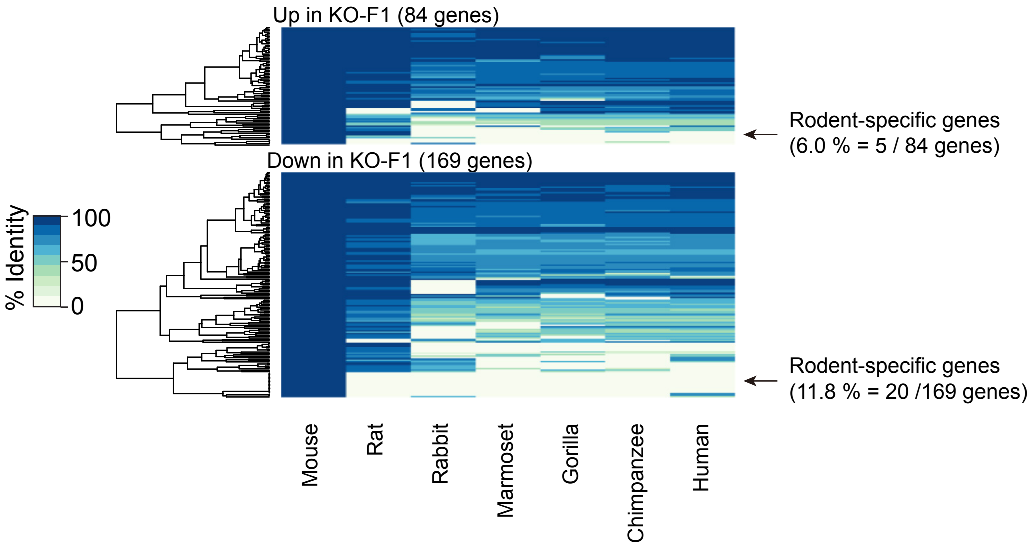

Supplementary Figure S5. Evolutionary feature of dysregulated genes in *Scml2-KO*-derived F1 spermatozoa

Heatmap showing percentage sequence identity for each dysregulated gene across 6 other species. Dysregulated genes in *Scml2*-KO-derived F1 spermatozoa exhibit low levels of DNA conservation.

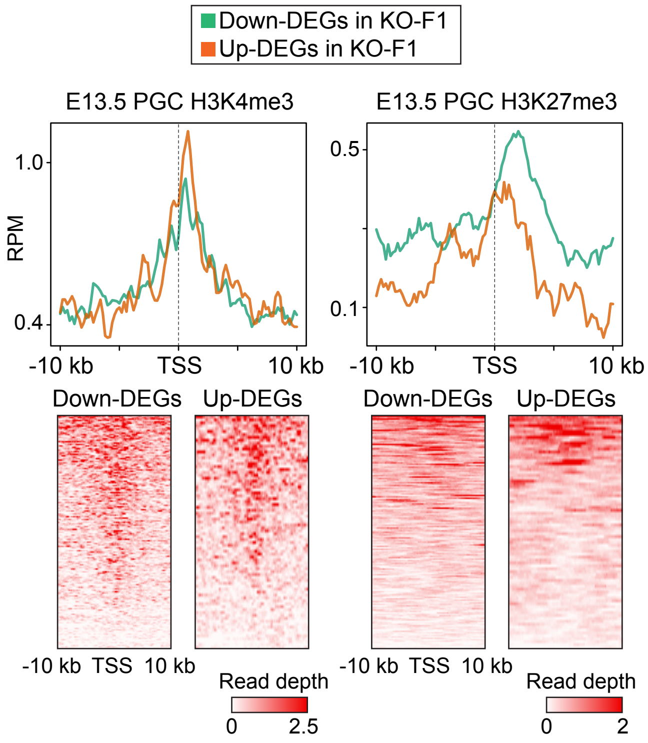

Supplementary Figure S6. Epigenetic landscapes of DEGs in *Scml2*-KO-F1 spermatozoa in primordial germ cells.

Average tag density plots and heatmaps of H3K4me3 and H3K27me3 wild-type E13.5 male PGC ChIP-seq reads around TSS (±10 kb) of down- and up-DEGs in *Scml2*-KO-F1 spermatozoa.

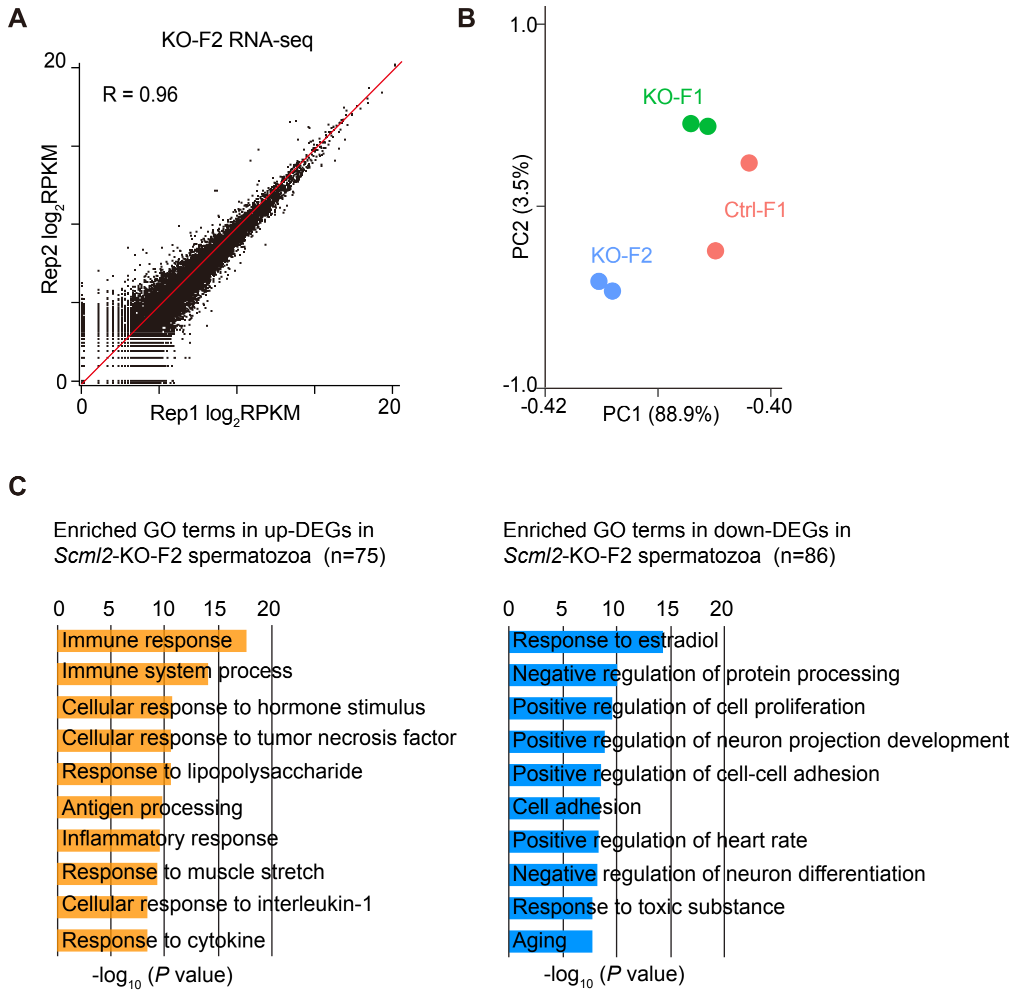

Supplementary Figure S7. RNA-seq analyses of *Scml2*-KO F2 sperm

(**A**) A scatter plot shows the correlation between biological replicates of RNA-seq reads at each 10kb bin. RNA-seq reads are shown in log2 RPKM values. Pearson correlation values (R) are shown.

(**B**) A scatter plot shows the PCA analysis among RNA-seq data of *Scml2*-KO F1 sperm (KO-F1), their control F1 (Ctrl-F1) sperm, and *Scml2*-KO F2 (KO-F2) sperm.

(**C**) Comparison of transcriptomes between Ctrl-F1 and *Scml2*-KO-F2 spermatozoa. Bar chart showing enriched GO terms for the up- and down-DEGs between Ctrl-F1 and *Scml2*-KO-F2 spermatozoa.

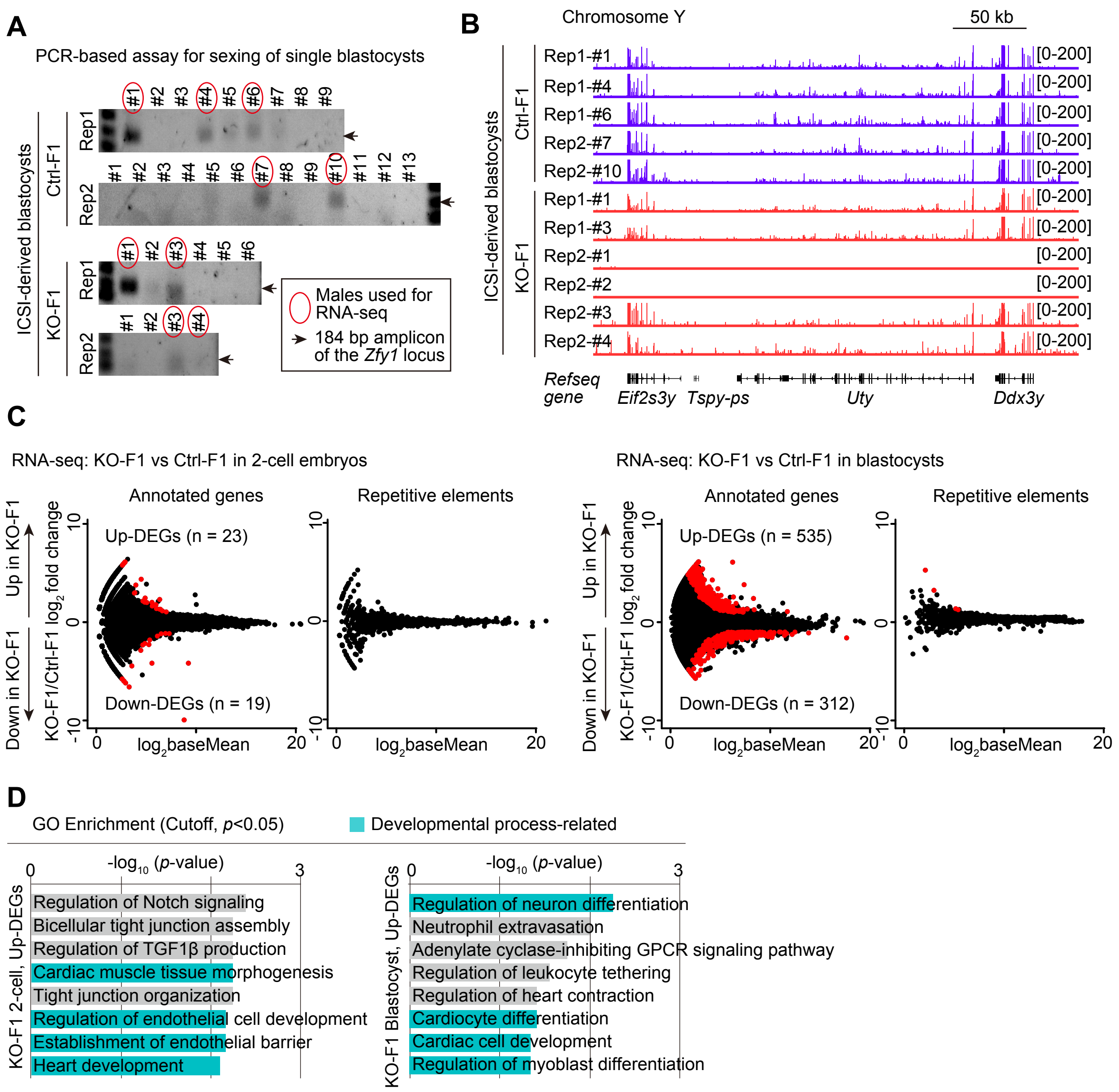

Supplementary Figure S8. RNA-seq analyses of ICSI-derived Ctrl-F1 and *Scml2*-KO-F1 embryos at the 2-cell and blastocyst stages.

(**A**) Agarose gel electrophoresis images of sex genotyping of single blastocysts sired by ICSI with Ctrl and *Scml2*-KO testicular sperm. The specific primer set targeted the *Zfy1* (zinc finger protein 1, Y-linked) locus and amplified a 184 bp product following PCR of DNA extracted from XY blastocysts but not from XX blastocysts. Red circles highlight XY blastocysts used for RNA-seq. Note that all blastocysts sired from Rep#2 of *Scml2*-KO spermatozoa were subjected to RNA-seq analysis because the PCR results were not clear as shown, and males were confirmed with the presence of the Y chromosome as shown in panel (**B**).

(**B**) Representative track view shows RNA-seq reads in ICSI-derived Ctrl-F1 and *Scml2*-KO-F1 blastocysts at a Y chromosomal region. The y-axis represents normalized tag counts for RNA-seq in each sample. Because there were almost no RNA-seq reads from *Scml2*-KO-F1 Rep2-#1 and -#2 blastocysts aligned to the Y chromosome, we judged their genetic sex as XX and excluded them from subsequent analyses.

(**C**) RNA-seq differential expression analysis: *Scml2*-KO-F1 versus Ctrl-F1 embryos at 2-cell and blastocyst stages; in total, 42 and 847 annotated gene transcripts evinced significant changes in expression in *Scml2*-KO-F1 2-cell and blastocyst embryos, respectively (red circle, fold change $\geq |2|$, P value < 0.01, binomial test). In contrast to annotated gene transcripts, only a few types of repetitive elements were differentially expressed in *Scml2*-KO-F1 blastocysts. Up-DEGs: upregulated differentially expressed genes; Down-DEGs: downregulated differentially expressed genes.

(**D**) Gene ontology (GO) analysis of Up-DEGs in *Scml2*-KO-F1 embryos, assessed by the Enrichr (https://maayanlab.cloud/Enrichr/). The GO terms with P values < 0.05 (Fisher's exact test) were defined as enriched. The ontological groups related to developmental processes were highlighted in turquoise.

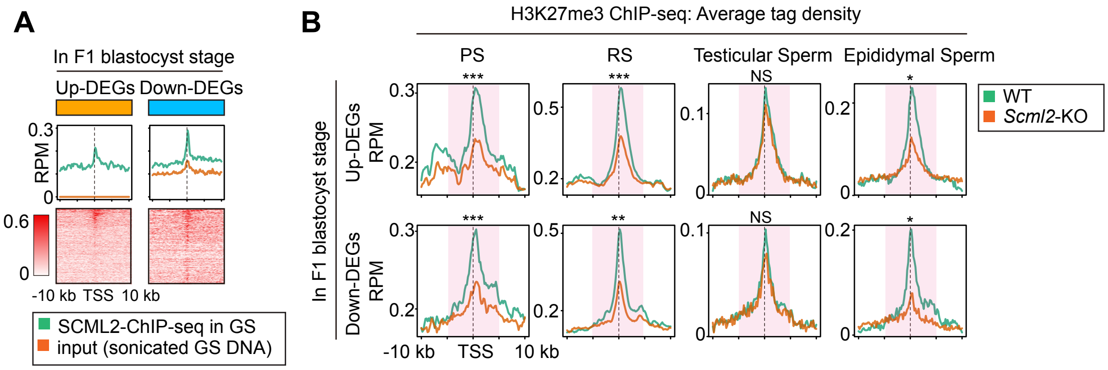

Supplementary Figure S9. Epigenetic landscapes of DEGs in *Scml2*-KO-F1 blastocysts in the male germline.

(**A**) Average tag density plots and heatmaps around TSS ±10 kb of SCML2 ChIP-seq reads in germline stem (GS) cells on up- and down-DEGs in *Scml2*-KO-F1 blastocysts.

(**B**) Average tag density plots of H3K27me3 around TSS ±10 kb of down- and up-DEGs in PS, RS, testicular sperm, and epididymal sperm from wild-type and *Scml2*-KO males. *P < 0.05; **P < 0.01; ***P < 0.001; NS, not significant; Wilcoxon rank-sum test with Bonferroni correction was performed around TSS (±5 kb) (shown with pink areas).

Supplementary Table S1

Full-term development of ICSI- and ROSI-embryos fertized with testicular sperm or round spermatids from wild-type and *Scml2* -KO mice

	No. of injected oocytes	No. of survived oocyte (%)	No. of 2-cell embryos (%)	No. of 1-cell arrest (%)	No. of fragmented (%)	No. of transferred embryos	No. of pups (%) [min-max]	Body weight (average ± SE)	Placental weight (average ± SE)
WT ICSI	144	76 (53)	63 (83)	8 (11)	5 (7)	63	7 (11) [0-14.6]	1.73 ± 0.05	0.10 ± 0.01
KO ICSI	277	187 (68)	146 (79)	22 (11)	19 (9)	146	9+7* (11) [4.8-19.0]	1.79 ± 0.04 #	0.14 ± 0.02
WT ROSI	108	71 (66)	64 (90)	5 (7)	2 (3)	64	5 (8) [0-13.5]	1.59 ± 0.03	0.12 ± 0.01
KO ROSI	301	203 (67)	191 (94)	6 (3)	6 (3)	191	7 (4) [2.4-4.7]	1.63 ± 0.07	0.14 ± 0.02

Two independent male mice were used for each experiment. ICSI = intracytoplasmic sperm injection, ROSI = round spermatid injection

* obtained by natural childbirth

# not contain natural birth's data

Supplementary Table S2
Preimplantation development of ICSI-embryos fertilized with testicular sperm from wild-type and *Scml2* -KO mice

Experiments to obtain brastocysts

	No. of injected oocytes	No. of survived oocyte (%)	No. of activated zygote (%)	No. of cultured embryos*	No. of 1-cell arrest (%)	No. of fragmented (%)	No. of 2-cell embryos (%)	No. of 4-8-cell embryos (%)	No. of morulae (%)	No. of blastcyst (%)
WT(#1)	217	155(71)	133(61)	133	13(10)	6(5)	69(52)	25(19)	8(6)	12(9)
WT(#2)	220	149(68)	104(47)	104	6(6)	3(3)	45(43)	36(35)	0(0)	14(13)
KO(#1)	219	177(81)	115(53)	92	18(20)	16(17)	25(27)	22(23)	3(3)	8(9)
KO(#2)	216	167(77)	120(56)	75	9(12)	8(10)	29(39)	24(32)	1(1)	4(5)

Two independent male mice were used for each genotype (#1 and #2).
* Not all of KO-F1 embryos were used for culture experiments, while all WT-F1 embryos were used for culture experiments.

Experiments to obtain 2-cell embryos

	No. of injected oocytes	No. of survived oocyte (%)	No. of activated zygote (%)	No. of cultured embryos	No. of 1-cell arrest (%)	No. of fragmented (%)	No. of 2-cell embryos (%)
WT(#1)	157	91(58)	85(93)	85	8(9)	8(9)	69(81)
WT(#2)	156	90(58)	72(80)	72	3(4)	5(7)	64(89)
KO(#1)	92	81(88)	74(91)	74	4(5)	12(16)	58(78)
KO(#2)	92	80(87)	69(86)	69	3(4)	5(7)	60(87)

Two independent male mice were used for each genotype (#1 and #2).
